# A case of traumatic urethral stricture complicated by periurethral abscess while awaiting delayed urethroplasty

**DOI:** 10.1002/iju5.12490

**Published:** 2022-05-30

**Authors:** Masayuki Shinchi, Akio Horiguchi, Eiji Takahashi, Fumihiro Kimura

**Affiliations:** ^1^ Department of Urology National Defense Medical College Saitama Japan; ^2^ Department of Urology Nishisaitama‐chuo National Hospital Saitama Japan

**Keywords:** periurethral abscess, urethral trauma, urine drainage

## Abstract

**Introduction:**

We report a case of bulbar urethral injury complicated by periurethral abscess due to inappropriate suprapubic catheter management.

**Case presentation:**

A 58‐year‐old man with bulbar urethral injury due to perineal trauma was referred to our hospital, and a suprapubic catheter was inserted for initial management. Although he was instructed to connect the catheter to the urine collection bag, he connected a plug to the catheter. As a result, he developed periurethral abscesses due to extravasated urine from the injured urethra, requiring percutaneous drainage and prolonging the time to definitive urethroplasty for the urethral stricture.

**Conclusion:**

It is essential that the suprapubic catheter be connected to a urine collection bag rather than a plug to keep the bladder as empty as possible and to minimize extravasation of the urine from the injured urethra.

**Abbreviations & Acronyms**
MRI: magnetic resonance imaging
PR: primary realignment
SPC: suprapubic catheter

**Keynote message**

Suprapubic catheter placement is ideal for the acute management of urethral injury. It is essential that the catheter be connected to a bag rather than a plug to keep the bladder as empty as possible and to minimize extravasation of the urine from the injured urethra.

## Introduction

The key to the initial management of bulbar urethral injury due to perineal trauma is prompt drainage of urine to prevent infection and minimize the damage to the injured urethra; suprapubic catheter (SPC) placement is the most ideal method for initial management.^1^ The SPC can be connected to a urinary collection bag or a connecting plug; however, no findings have been reported on which method is more appropriate in patients with urethral injury. Herein, we report a case of a patient with urethral injury who developed a periurethral abscess due to inappropriate management of an SPC.

## Case presentation

A 58‐year‐old man slipped in the bath and hit his perineum on the edge of the bathtub. After the injury, he experienced bleeding from the external urethral meatus, urinary retention, and hematoma in the scrotum. He was referred to our hospital by his local physician. Retrograde urethrography showed no extravasation of the contrast medium, but the urethral lumen was completely obliterated at the proximal bulbar urethra due to hematoma, which strongly suggested bulbar urethral injury. He was admitted, and a 14‐French SPC was placed under local anesthesia for urine drainage. The urethral bleeding and scrotal hematoma were resolved conservatively with rest and local compression, and the patient was discharged after being instructed to connect the SPC to a urine collection bag to keep the bladder as empty as possible.

**Fig. 1:**
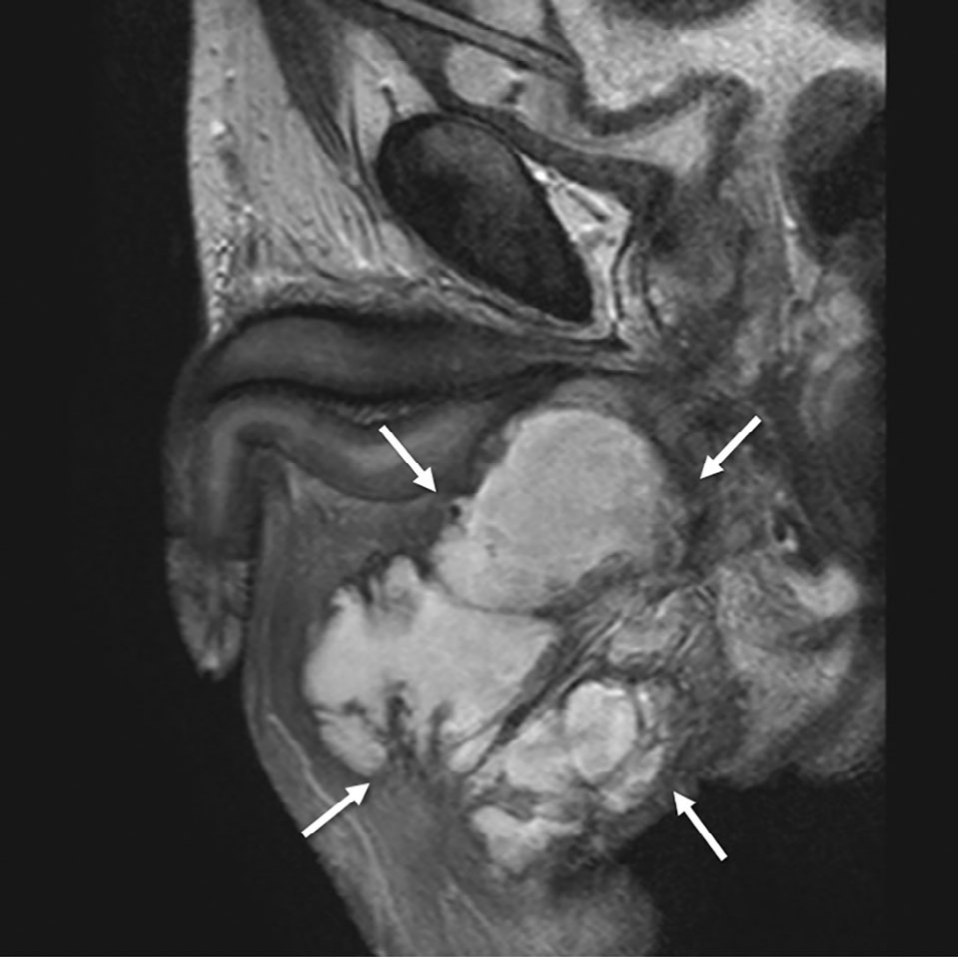
Sagittal T2‐weighted imaging showing extensive periurethral abscess in the perineum (arrows).

**Fig. 2:**
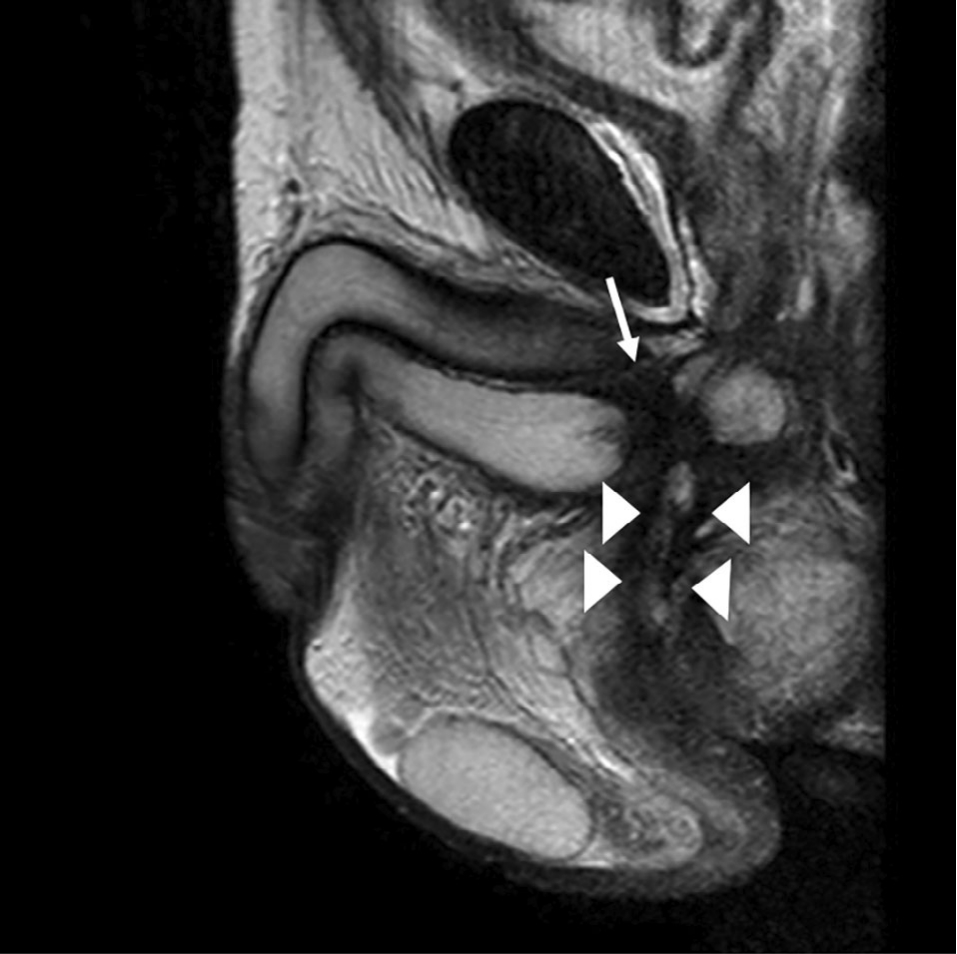
Sagittal T2‐weighted imaging showing extensive spongiofibrosis (arrow) and a periurethral fistula extending to the perineum (arrowheads).

**Fig. 3:**
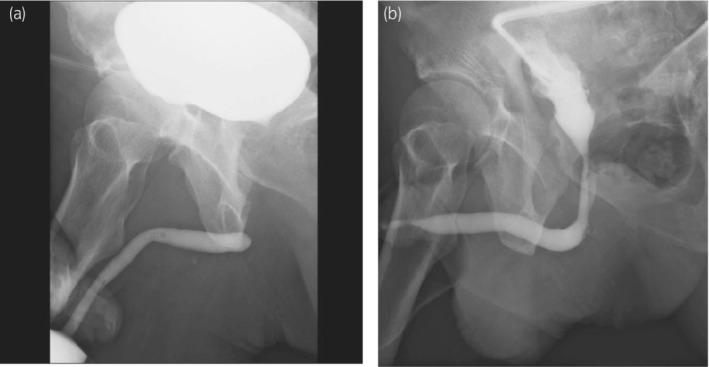
(a) Preoperative and (b)postoperative urethrography.

Thereafter, he visited us for catheter replacement 4 weeks after discharge and requested the use of a plug instead of a urine collection bag because it was inconvenient for his return to work. One week after using the plug, he visited our hospital with a swollen scrotum and perineum and high fever. T2‐weighted magnetic resonance imaging (MRI) revealed findings suggestive of an abscess extending from the bulbar urethra to the perineum and scrotum (Fig. 1). He was advised to stop using the plug and to undergo percutaneous drainage of the abscess. It was found that the injured urethra was completely obstructed, and delayed urethroplasty was necessary, but it had to be postponed. T2‐weighted MRI 6 months after abscess formation showed severe spongiofibrosis in the proximal bulbar urethra and an urethrocutaneous fistula (Fig. 2). Ten months after the urethral injury, the patient underwent excision and primary anastomosis for the traumatic bulbar urethral stricture. Currently, there has been no report of stricture recurrence (Fig. 3).

## Discussion

It is likely that complications derived from urethral strictures would significantly impact patient quality of life. Complications during the waiting period for definitive urethroplasty have been reported with approximately 16% of patients experiencing complications while waiting for urethroplasty.^2^

SPC placement and primary realignment (PR) are the two urine drainage methods for the initial management of urethral injury. However, recently, some medical professionals have argued that PR carries the risk of creating a pseudo‐passage or fistula, making it difficult to perform delayed urethroplasty.^3^ In our study of the relationship between the initial treatment and subsequent clinical courses in patients with traumatic bulbar urethral stricture, those who underwent PR had complicated secondary urethral strictures, thus prolonging the time to definitive treatment.^4^ In contrast, SPC is a simple procedure without any risk of further damage to the urethra; hence, we exclusively chose SPC placement.

SPC placement in an acute setting should prevent urine extravasation from the injured urethra and subsequent infection; however, in this case, a severe abscess requiring drainage had developed. Since the abscess developed when the patient started using the plug instead of the urine collection bag, it can be inferred that the plug use was a contributing factor for the abscess occurrence. There are only some reports discussing whether the urine collection bag or the plug is better in patients with urinary catheters.^5^, ^6^ Most of them reported that there was no difference in the incidence of bladder spasm or urinary tract infections between these two methods, but the patients preferred to use the plug because of the associated ease of movement and appearance. However, these studies were conducted in cohorts of patients with chronic diseases, such as benign prostatic hyperplasia and neurogenic bladder, and only few examined catheter management after urethral injury. To the best of our knowledge, this is the first report of a case of periurethral abscess in the subacute to chronic phase 4 weeks after urethral injury.

Generally, damaged tissues undergo three phases of healing: inflammation, proliferation, and remodeling.^7^ It has been reported that the healing process after urethral injury requires at least 3 months.^8^ In this case, the urethral tissue had not yet healed, and the injured area may still have been vulnerable 1 month after the injury. This patient had been complaining of a sensation of urine flowing into the injured proximal urethra during defecation since the creation of the SPC. The cause of urine extravasation is the urinary reflex caused by catheter stimulation. Since the patient with a plug has a larger volume of urine in the bladder, the amount of extravasation when the urinary reflex occurs is greater and more likely to lead to complications. It is speculated that the change from the urinary collection device to the plug increased the amount of urine stored in the bladder, resulting in increased urine extravasation into the unhealed urethra. Thus, it is extremely important to connect the SPC to a urine collection bag and prevent catheter obstruction for at least 3 months after urethral injury until the injured area heals completely and to keep the bladder as empty as possible to prevent urine extravasation.

## Author contributions

Masayuki Shinchi: Conceptualization; writing – original draft. Akio Horiguchi: Project administration; supervision; validation. Eiji Takahashi: Investigation; supervision. Fumihiro Kimura: Supervision.

## Conflict of interest

The authors declare no conflict of interest.

## Institutional reviewer board approval and the approval number

N/A

## Informed consent

All informed consent was obtained from the subject.

## Registry and the registration no. of the study/trial

N/A
